# Methods of Assessment of Metal Contamination in Bottom Sediments (Case Study: Straszyn Lake, Poland)

**DOI:** 10.1007/s00244-019-00662-5

**Published:** 2019-08-19

**Authors:** Eliza Kulbat, Aleksandra Sokołowska

**Affiliations:** Faculty of Civil and Environmental Engineering, Gdansk University of Technology, Gdańsk, Poland

## Abstract

The concentrations of six metals (Zn, Cu, Pb, Ni, Cr, and Cd) were investigated in bottom sediments of Straszyn Lake (North Poland). This study was designed to determine a total content of metals and to assess their mobility and bioavailability. The sequential extraction was used to fractionate metals into five fractions: exchangeable, bound to carbonates, bound to Fe–Mn oxides, bound to organic matter, and residual. The evaluation of sediments contamination degree by metals was performed by applying the geochemical quality guidelines, the pollution load index, and the geo-accumulation index (*I*_geo_). The assessment based on these methods demonstrated that sediments were polluted with Cr and the sediments quality guidelines confirmed these results. Moreover, the average concentrations of Cu, Ni, and Cr were respectively 3.4, 3.9, and 21.2 times higher than their background values. According to ecological risk index and risk assessment code Cd was the most important factor affecting the ecological environment of the Straszyn Lake. The metal speciation analysis demonstrated that the mean percentage of metals in the exchangeable and carbonate fractions decreased in the following order: Cd (59.1%) > Zn (19.8%) = Ni (19.8%) > Pb (16.6%) > Cu (3.3%) > Cr (2.7%). The very strong correlation calculated between all the metals indicated their common origin.

Metals in water sediments are partially bound in the structure of minerals which are relatively resistant to weathering (e.g., in feldspar, heavy minerals) and do not pose a threat to the biosphere, and partially present in newly formed chemical compounds (sulphides, carbonates, oxides) or in forms absorbed by clay minerals, organic matter, or hydrated iron hydroxides (Bojakowska and Sokołowska 1998). The granulometric composition of sediments affects the accumulation of metals from the water phase and the degree of their binding on the surface of the solid phase. The smallest granulometric fractions of sediments have a high capacity to absorb metal cations on the surface and thus bind metals in sediments (Szarek-Gwiazda et al. 2011; Adiyiah et al. 2014; Strzebońska et al. 2015; Baran et al. 2016). The process of metal sorption on suspension particles proceeds with varying intensity depending on the type of metal (Wang et al. 2016). Metals accumulated in bottom sediments may be released to the water depth due to various chemical and biochemical processes occurring in bottom sediments. Hydrological factors, microbial activity, and physicochemical conditions in the water-sediments interface may affected metal remobilization (Calmano et al. 1993; Förstner 2004; Yang et al. 2009; Guven and Akinci 2013). The components of bottom sediments, including metals, also can be taken directly from sludge by benthic organisms. To assess the dangers arising from the presence of metals in bottom sediments of water reservoirs, it is necessary to know not only their total content, but also speciation, because metal bioavailability is dependent on the forms that they adopt in a given element of the environment (Fernandes and Nayak 2014; Baran and Tarnawski 2015; Pejman et al. 2015; Wojtkowska et al. 2016; Ke et al. 2017). Determination of metal forms in sediments also makes it possible to assess the potential migration of metals from sediments to water and the potential toxicity of metals (Sundaray et al. 2011; Pejman et al. 2015; Palleiro et al. 2016). The assessment of the degree of contamination of bottom sediments with metals is particularly important in the case of reservoirs constituting drinking water resources.

In Poland, currently no legal provisions allow assessment of the level of pollution of bottom sediments with metals. The only legal act that was in force until 2013 was the *Regulation of the Minister of Environment of 16 April 2002 on the types and concentrations of substances that cause spoilage is contaminated*, specifying criteria for the following metals: arsenic, chromium, zinc, cadmium, copper, nickel, and mercury. Research on the quality of bottom sediments is performed by the State Environmental Monitoring as part of inland water monitoring. The research is based on the geochemical classification of river and lake sediments developed by the Polish Geological Institute (Bojakowska and Sokołowska 1998). In this classification, the settlements were divided into 3 classes, taking into account the content of 11 metals in relation to the adopted thresholds. Both methods of assessing the degree of sediment pollution are based solely on the assessment of the total content of metal. Many authors use geochemical criteria to assess the degree of pollution of bottom sediments by metals, in which the total content of metals in sediments is assessed in relation to the geochemical background value. These criteria are primarily the geo-accumulation index *I*_geo_ (Müller 1969; Farkas et al. 2007; Zahra et al. 2014; Ke et al. 2017; Duncan et al. 2018) and the pollution load index (PLI) (Wilson et al. 2008; Kuriata-Potasznik et al. 2016; Alshahri and El-Taher 2018). The results of such an assessment depend to a large extent on the assumed geochemical background values. To assess the level of contamination safe for organisms, however, it is necessary to apply ecotoxicological criteria and to assess the bioavailability of metals based on speciation analysis. The often-used ecotoxicological criteria are sediments quality guidelines (SQGs) (MacDonald et al. 2000; Long 2006; Ke et al. 2017), based on two threshold metal levels above which toxic effects of metals on organisms can be observed at different frequencies. In turn, the potential ecological risk index for trace elements (PERI) is an indicator that enables assessment of potential ecological risk, which takes into account the toxicology of metals (Hakanson 1980). The primary objectives of this study were: (1) to determine the concentrations of metals, including Cd, Cu, Cr, Ni, Pb, and Zn, in the surface sediments of Straszyn Lake; (2) to investigate the mobility of metals in sediments by using chemical fractionation; (3) to assess the potential ecological risk of metals in sediments using PLI, *I*_geo_, SQGs, PERI, and RAC.

## Materials and Methods

### Study Area

The study was conducted in Straszyn Lake, which is located on the Radunia River of northern Poland and lies at the eastern edge of the Kashubian Lakes District. This reservoir was constructed in 1910, primarily to produce energy. A small hydroelectric power plant with a capacity of 2450 kW is situated here. The catchment area of the Radunia River equals to 837 km^2^, and as much as 67.6% of the area is agricultural land. Intensive farming is performed in this area, but it also is a tourist destination, in particular the west part of Radunia’s catchment (report of GIEP 2013). The total volume of the Straszyn Reservoir is 3 mLn m^3^, its surface area is 0.75 km^2^, and its average retention time is 170 h (Best et al. 1997). The reservoir is 3-km long. Since 1986, the main function of this reservoir is the provision of drinking water to several districts of Gdańsk.

### Sample Collection

**Fig. 1:**
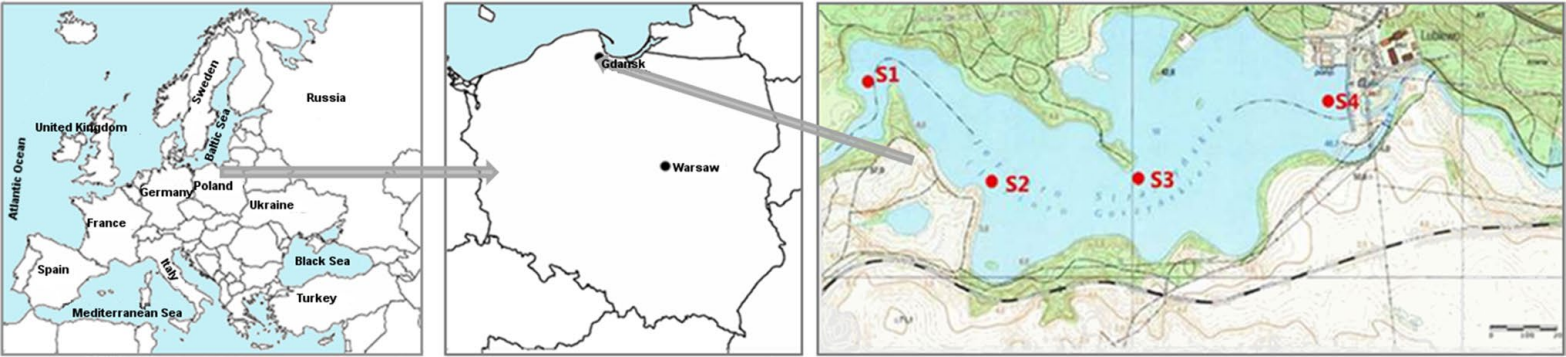
Location of Straszyn Lake in Poland and positions of sampling sites

The sediment sampling locations were chosen along the main stream of the reservoir. The ordinal numbers of the points increase from the inflow of the Radunia River to the water intake at the dam. The sampling locations are shown in Fig. 1.

The collection of the superficial sediment samples was performed on 8 occasions between May and October 2013 at intervals of approximately 20 days. Sediment samples with thickness of 10–15 cm were collected using a KC Kajak sediment core sampler (KC Denmark Research Equipment, Denmark) and stored in acid-washed plastic containers. After collection, the samples were air-dried and sieved using a 2-mm plastic sieve to remove large pieces of detritus.

### Sample Pretreatment and Chemical Analysis

The sediment samples were analyzed in terms of grain size, pH, organic matter content, and the concentrations of the selected metals (Zn, Cu, Pb, Ni, Cr, and Cd). The grain-size distributions of the sediment samples were measured using the wet sieving method (sand: 1–0.05 mm, clay silty: 0.05–0.002 mm, clay < 0.002 mm) and an Analysette 28 Image Sizer. The content of organic matter was determined using the loss-on-ignition (LOI) technique at 605 °C. To measure the concentrations of the metals, the sediment samples were dried at 105 °C to constant weight and sieved through a 0,2 mm sieve. To determine the contents of metals the sediment samples were extracted with aqua regia (HCl:HNO_3_ = 3:1) using a Büchi K-438 digestion system (Sastre et al. 2002). All of the concentrated acids used for the experiments were supra pure (Merck) quality. The speciation analysis of metals was performed using the five-step Tessier’s sequential procedure (Tessier et al. 1979):

- F1: exchangeable, extractable with 8 mL of MgCl_2_ (1 mol/L, pH 7), for 1 h, at room temperature, with continuous agitation;
- F2: bound to carbonates, extractable with 8 mL of NaOAc (1 mol/L, adjusted to pH 5.0 with HOAc), for 8 h, at room temperature, with continuous agitation;
- F3: bound to Fe–Mn oxides, extractable with 20 mL of NH_2_OH–HCl (0.04 mol/L) in 25% (v/v) HOAc, for 8 h, at 96 ± 3 °C, with occasional agitation;
- F4: bound to organic matter, extractable with 3 mL of HNO_3_ (0.02 mol/L) and 5 mL of 30% H_2_O_2_ adjusted to pH_2_ with HNO_3_, the mixture was heated to 85 ± 2 °C for 2 h with occasional agitation. A second 3 mL of 30% H_2_O_2_ (pH_2_) was then added, and the sample was heated again to 85 ± 2 °C for 3 h with intermittent agitation. After cooling, 5 mL of NH_4_OAc (3.2 mol/L) in 20% (v/v) HNO_3_ was added and the sample was diluted to 20 mL and agitated continuously for 30 min;
- F5: residual, extractable with a mixture of concentrated acids in a ratio of 3:1 of HCl:HNO_3_ (aqua regia) for 3 h according to the procedure described for total metal analysis.

Standard solutions for Zn, Cu, Pb, Ni, Cr, and Cd were prepared from the 1000 mg/L Merck Certipur^®^ standard solutions. All solutions were prepared with deionized water obtained from a Millipore Elix-3 water purification system. The analysis of blank reagents and reference materials was performed along the same experimental procedure. The lab glassware was precleaned by soaking in 10% HNO_3_ and rinsing with deionized water prior to use.

The metal concentrations in the sediments were measured using a Vario 6 flame atomic absorption spectrometer (F-AAS) with an air/acetylene flame that was equipped with a single-element hollow cathode lamp and a deuterium lamp to enable background corrections. The reported detection limits were 0.012 mg/L for Zn, 0.04 mg/L for Cu, 0.015 mg/L for Pb, 0.011 mg/L for Ni, 0.020 mg/L for Cr, and 0.017 mg/L for Cd. All of the measurements were performed three times, and the mean values are reported. The replicate measurements agreed within < 5%. The accuracy of the analytical method was assessed using a sedimentary reference material (river clay sediment LGC6139). The measured contents of the metals in the analytical standard are given in Table 1.

**Table 1:** Measured content of metals in the analytical sedimentary reference material LCG6139

Metal mg/kg	Measured content of metal in the analytical standard	Certified metal content (LGC6139)
Zn	510 ± 3	513
Cu	90 ± 0.8	92
Pb	162 ± 1.7	160
Ni	39 ± 0.3	38
Cr	79 ± 0.8	80
Cd	2.38 ± 0.02	2.3

During the metal analyses, calibration solutions, blanks, and standards were re-run every 12 samples. The mean measured values for the blanks were below the reported detection limits.

## Assessment of Sediment Contamination

### Geochemical Background and Geochemical Quality Guidelines

The simplest method of assessing the content of metals in bottom sediments is to compare it to the value of the geochemical background (Baran et al. 2011). Due to the regional (limited by area) range of the geochemical background (Gałuszka 2007), the data given in the Geochemical Atlas of Pobrzeże Gdańskie were adopted as the basic values of it (Lis and Pasieczna 1999). Parallel calculations were made by using the background values provided by Bojakowska and Sokołowska (1998), accepted as a geochemical background by many authors analyzing the quality of bottom sediments in Poland (Gierszewski 2008; Skorbiłowicz and Skorbiłowicz 2009; Szalińska et al. 2010; Skorbiłowicz 2014; Baran et al. 2016; Kuriata-Potasznik et al. 2016; Podlasińska and Szydłowski 2017; Ciazela et al. 2018). The assessment of sediment contamination with metals was based on the Polish geochemical classification of bottom sediments (Bojakowska 2001) (Table 2).

**Table 2:** Geochemical quality classes of bottom sediments and geochemical background in Poland

Parameters	Zn	Cu	Pb	Ni	Cr	Cd
Geochemical quality classes^a^ (µg/g d.m.)						
Class I	125	20	30	16	50	0.7
Class II	300	100	100	40	100	3.5
Class III	1000	300	200	50	400	6
Class IV	> 1000	> 300	> 200	> 50	> 400	> 6
Geochemical background^a^ (µg/g d.m.)						
	48	6	10	5	5	0.5
Geochemical background^b^ (µg/g d.m.)						
	41	5	11	4	7	0.5

^a^Bojakowska (2001)

^b^Lis and Pasieczna (1999)

### Pollution Load Index

To assess the extent of pollution by metals in bottom sediments, the simple method based on pollution load index (PLI) proposed by Tomlinson et al. (1980) was applied. PLI is defined as:

$$ {\text{PLI}}\;{\text{for}}\;{\text{a}}\;{\text{site }} = { (}C_{{f_{1} }} \times \, C_{{f_{2} }} \times \cdots \times \, C_{{f_{n} }} )^{1/n} $$

$$ {\text{PLI}}\;{\text{for}}\;{\text{ a}}\;{\text{zone }} = {\text{ (PLI}}_{1} \times {\text{ PLI}}_{2} \times \, \cdots \times {\text{ PLI}}_{n} )^{1/n} $$

where *C*_f_ is the contamination factor for individual metals: *C*_f_ = *C*_*i*_*/C*_0_, *C*_*i*_ is the concentration of metal *i* in sediment, *C*_0_ is the background value of the metal in the study area, *n* = number of metals (PLI for site), or *n* = the number of sites (PLI for zone).

According to Zhu et al. (2016), the PLI was classified as: 0 < PLI ≤ 1 unpolluted; 1 < PLI ≤ 2 moderately to unpolluted; 2 < PLI ≤ 3 moderately polluted; 3 < PLI ≤ 4 moderately to highly polluted; 4 < PLI ≤ 5 highly polluted; PLI > 5 very highly polluted. Additionally the contamination degree (*C*_d_) was calculated based of the sum of all contamination factors (Hakanson 1980; Harikumar et al. 2009; Bhuiyan et al. 2010). The classification for contamination factor (*C*_f_) and contamination degree (*C*_d_) is presented in Table 3.

**Table 3:** Classification for contamination degree

Contamination factor *C*_f_	Contamination degree *C*_d_	Classification
< 1	< 8	Low
1–3	8–16	Moderate
3–6	16–32	Considerable
≥ 6	≥ 32	Very high

### Geo-accumulation Index

To establish the contamination status of the sediments of the Straszyn Reservoir, the geo-accumulation index (*I*_geo_) was calculated for the samples, and they were classified according to their *I*_geo_ values (Müller 1969):

$$ I_{\text{geo}} = \, \log_{2} \left( {C_{n} /1.5B_{n} } \right) $$

*C*_*n*_, concentration of element “*n*” in the < 0.002-mm (clay) fraction; *B*_*n*_, background value for this element for this size fraction in river sediments

The factor of 1.5 accounted for possible variations in the background data.

The *I*_geo_ consists of seven grades, of which the highest grade reflects a 100-fold enrichment relative to the background value. The geo-accumulation classes and the corresponding contamination intensity listed by Förstner et al. (1993) were used (Table 4).

**Table 4:** Geo-accumulation index (*I*_geo_) and contamination levels

Sediment *I*_geo_ contamination	Levels	Pollution degree
< 0	0	Practically unpolluted
0–1	1	Unpolluted to moderately polluted
1–2	2	Moderately polluted
2–3	3	Moderately to strongly polluted
3–4	4	Strongly polluted
4–5	5	Strongly to very strongly polluted
> 5	6	Very strongly polluted

### Sediment Quality Guidelines

A potential threat to organisms associated with the presence of metals in bottom sediments was assessed with the help of numerical sediments quality guidelines (SQGs) (MacDonald et al. 2000; Ke et al. 2017). This is an ecotoxicological criterion based on two threshold metal contents: Threshold Effect Concentration (TEC) and Probable Effect Concentration (PEC). In the case of metal concentrations reaching values above TEC, their toxic effects on organisms can be observed (rarely), while above PEC there occur often adverse biological effects. The TEC and PEC values are presented in Table 5.

**Table 5:** Metal sediment quality guideline values (µg/g)

	Zn	Cu	Pb	Ni	Cr	Cd
TEC	121	31.6	35.8	22.7	43.4	0.99
PEC	459	149	128	48.6	111	4.98

The consensus-based PECQ can be used to reliably predict toxicity of sediments (Ingersoll et al. 2001). The average PEC quotient (PECQ) for the six tested trace elements was analyzed. For each sediment sample, the PECQ average was the average of the concentration ratio of each element to the corresponding PEC. PECQ values < 0.5 indicate that sediment samples were not toxic (low potential toxicity to benthic fauna), whereas sediment samples with PECQ > 0.5 were toxic, which indicates toxicity of sediment samples and high potential risk for the bottom fauna (MacDonald et al. 2000; Niu et al. 2009).

### Potential Ecological Risk Index

To assess the complex potential ecological risk caused by the presence of metals in sediments, the potential ecological risk index (PERI) was used. Hakanson (1980) introduced this method taking into account the toxicology of metals and assessing the potential ecological risk caused by general levels of pollution. The PERI was calculated as follows (Hakanson 1980; Ke et al. 2017):

$$ {\text{PERI }} = \sum\limits_{i = 1}^{n} {E_{r}^{i} } $$

The potential ecological risk factor of a chosen metal (*E*_*r*_^*i*^) is defined as:

$$ E_{r}^{i} = T_{r}^{i} \times \, C_{f}^{i} = \, T_{r}^{i} \times \, (C_{i} /C_{0} ) $$

where *C*_*f*_^*i*^ is the contamination factor of the trace elements, *C*_*i*_ is the concentration of metal *i* in sediment, and C_0_ is the background value of the metal in the study area. *T*_*r*_^*i*^ is the biological toxicity factor of an individual element, which was determined for Cu = Pb = Ni = 5, Zn = 1, Cr = 2, and Cd = 30 (Hakanson 1980; Guo et al. 2010; Suresh et al. 2011; Baran et al. 2016; Ke et al. 2017). The terminology used by Hakanson (1980) to describe *E*_*r*_^*i*^ and PERI is displayed in Table 6.

**Table 6:** Classification of PERI

Assessment criterion	Grades of potential ecological risk				
	Low	Moderate	Considerable	High	Very high
*E*_*r*_^*i*^	< 40	40–80	80–160	160–320	≥ 320
PERI	< 150	150–300	300–600	≥ 600	

### Risk Assessment Code

The risk assessment code (RAC) was applied in the study to assess the risk and mobility of the nonstable chemical fraction of metals (Singh et al. 2005; Baran and Tarnawski 2015; Ke et al. 2017). This classification is based on the percentage of metal in the exchangeable and carbonate fractions (Perin et al. 1985). The RAC is tabulated in Table 7.

**Table 7:** Risk assessment code

Risk	Metal in carbonate and exchangeable fractions (%)
No risk	< 1
Low risk	1–10
Medium risk	11–30
High risk	31–50
Very high risk	51–75

## Results and Discussion

### Physicochemical Properties of Bottom Sediments

The pH, grain size, and organic matter content of bottom sediments in Straszyn Lake are presented in Table 8. The sediments were characterized by pH ranging from neutral to slightly alkaline (6.9–7.6). The organic matter content (expressed as LOI) was observed between 4.5% and 18.4%. Silty clay fraction dominated except the station situated in the inlet zone. At this station higher amount of sand was found (up to 47%).

**Table 8:** Basic properties of bottom sediments

Parameters	Grain size (%)			pH	Organic matter (%)
	Sand	Clay silty	Clay		
Minimum	0	49	3.6	6.9	4.5
Maximum	47	92	8.2	7.6	18.4

### Concentrations of Metals in the Sediment of the Study Area

Table 9 summarizes the descriptive statistics related to the metal concentrations in sediment from Straszyn Lake.

**Table 9:** Descriptive statistics of metal concentrations in the Straszyn Lake sediments

Parameters	Zn	Cu	Pb	Ni	Cr	Cd
	µg/g d.m.					
Mean	55.19	17.08	21.73	15.56	148.2	0.72
Median	63.07	20.93	23.73	19.56	172.7	0.69
SD	24.99	8.25	12.23	9.14	81.18	0.72
Range	14.69–92.47	2.34–25.65	1.26–49.20	0.28–26.15	14.36–260.9	0.25–1.70
CV%	45	48	56	59	55	51

**Fig. 2:**
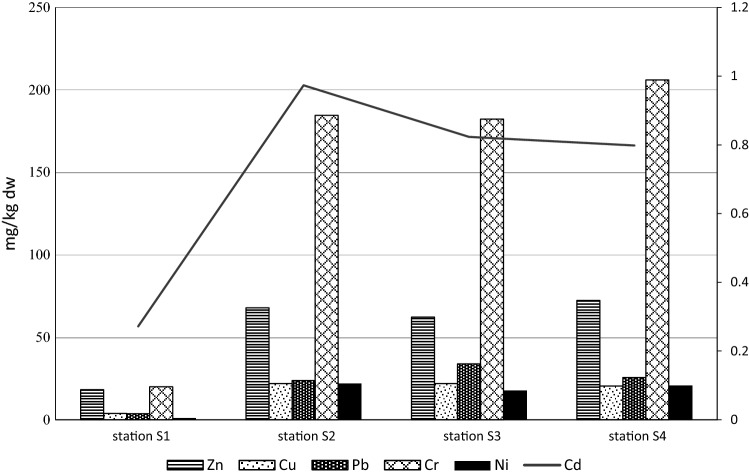
Spatial distribution of metals in the sediments (mean concentrations)

Metal concentration in the sediments ranged from 14.69 to 92.47 µg Zn/g d.m., from 2.34 to 25.65 µg Cu/g d.m., from 1.26 to 49.20 µg Pb/g d.m., from 0.28 to 26.15 µg Ni/g d.m., from 14.36 to 260.9 µg Cr/g d.m., and from 0.25 to 1.70 µg Cd/g d.m. (Table 9). The computed coefficients of variation (CV) for individual elements visibly exceeded 10% and were as follows: Zn—45%, Cu—48%, Pb—56%, Ni—59%, Cr—55%, Cd—51%. Thus, significant differences in metal concentrations in the sediments were observed. The lowest concentrations of all analyzed metals were characterized by samples taken from site 1, situated in the inlet zone, with high content of sand (Fig. 1). Spatial distribution of metals in the sediments was similar (Fig. 2).

Comparison of the mean values of metal concentrations with those in other reservoirs and lakes in Poland are listed in Table 10. The mean concentrations of Zn (55.19 µg/g), Pb (21.73 µg/g), and Cd (0.72 µg/g) in sediments of Straszyn Lake were similar to or lower than sediment from the other reservoirs. The mean concentrations of Cu (17.08 µg/g) and Ni (15.56 µg/g) were higher than for Byszyno Lake (respectively 8.6 µg/g and 2.7 µg/g) (Podlasińska and Szydłowski 2017) and Goreckie Lake (12 µg/g and 13 µg/g) (Zerbe et al. 1999) only. However, sediment from the Straszyn Lake was considerably greater in Cr concentration than the most of all the reservoirs presented in Table 10 data.

**Table 10:** Metal concentrations in sediment samples from Straszyn Lake and other selected lakes from the references

Location	Zn	Cu	Pb	Ni	Cr	Cd	References
	µg/g						
Straszyn Lake, north Poland	55.19	17.08	21.73	15.56	148.23	0.72	Present study
Goreckie Lake, central Poland	64	12	47.5	13	9.7	3.4	Zerbe et al. (1999)
Wloclawek reservoir, central Poland	158.0–650.0	32.0–55.8	35.9–68.8	8.4–33.5	29.0–330.0	Trace–12.3	Gierszewski (2008)
Chancza reservoir south Poland	61.50–212.0	6.50–89.60	13.90–43.15	5.05–26.90	5.25–30.20	Trace–0.86	Baran et al. (2011)
Rybnik reservoir, south Poland	79.74–1796	33.50–1506	35.69–136.8	3.29–68.75	2.92–132.7	0.10–15.67	Baran et al. (2016)
Symsar Lake, north Poland	110–130	14.56–22.36	45.65–118.96	26.03–34.75	8.68–19.76	0.36–0.69	Kuriata-Potasznik et al. (2016)
Byszyno Lake, north Poland	51.6	8.6	27.4	2.7	10.3	0.7	Podlasińska and Szydłowski (2017)

### Assessment of Sediment Contamination and Ecological Risk

#### Geochemical Background and Geochemical Quality Classes

Comparison of the average values of metals with the background values given by Lis and Pasieczna (1999) for Gdańsk Region (Table 2) showed that all the averages were higher. In particular, Cu, Ni, and Cr were respectively 3.4, 3.9, and 21.2 times greater than the background values. However, when comparing the average values of metals with the background values given by Bojakowska and Sokołowska (1998) for Poland (Table 2), the results indicated that these differences were varied: Cu, Ni, and Cr were respectively 2.8, 3.1, and 29.6 times greater than the background values. This indicates that human activities directly affect the concentration of Cu, Ni, and Cr in sediment. The mean concentration of metals exceeding background level was found for both values of the background in the order: Cr > Ni > Cu > Pb > Cd > Zn.

The assessment of sediment contamination with metals was based on Bojakowska’s geochemical quality classes of bottom sediments (2001) (Table 2). The results showed that because of concentration of Zn, Cu, Pb, Ni, Cr, and Cd respectively 100%, 46.9%, 78.1%, 31.3%, 25.0%, and 56.3% of sediment samples were uncontaminated (class I). The other samples of sediments were moderately contaminated (class II), except Cr: 75.0% of samples were classified as class III (contaminated sediments).

#### Pollution Load Index

For the PLI calculations, the data according to Lis and Pasieczna (1999) were used as the geochemical background values. Analyzes of metal content given by Lis and Pasieczna (1999) were made for more than 180 samples of bottom sediments of lakes in the research region, according to the methodology which was used in this work: aqua regia dilution extraction of sediment fractions < 0.2 mm and determination of metals according to ASA method. Methodology of designations (methods of sampling, sample preparation, e.g., screening, digestion) is important to settle the geochemical background and to assess the quality of sediments (Gałuszka 2007, Dung et al. 2013). Therefore, taking the value of the local geochemical background, determined by the same method, seems to be the best solution.

**Fig. 3:**
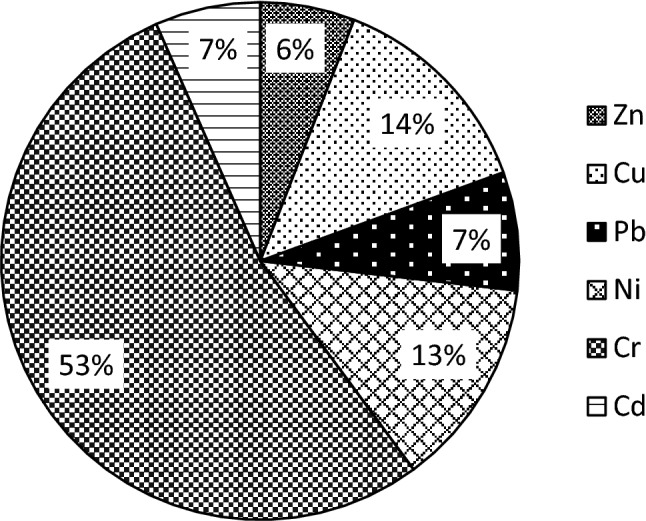
Proportion of metals in the contamination degree of bottom sediments of Straszyn Lake

The calculated PLI values of metals in sediments are summarized in Table 11. The mean PLI values ranged from 0.61 at the station S1 (the inlet zone) to 3.18 at the station S2; however, the PLI values at the stations S2–S4 were similar. The PLI value for Straszyn Lake calculated as PLI for a zone is 2.09, but if calculated for sites located in the middle of the reservoir and at the dam only (the stations S2–S4), it is clearly higher and reaches the value of 3.15. According to the PLI classification (Zhu et al., 2016), settlements at the station S1 should be classified as unpolluted (PLI < 1), but for all the other locations from moderately to highly polluted (3 < PLI ≤ 4), as well as the PLI value for the zone (the stations S2–S4) = 3.15. The contamination factors value analysis (*C*_f_) indicates that Cr is the metal, which has a significant effect on sediment pollution of Straszyn Lake. The contamination factor *C*_f_ calculated for Cr ranged from 2.86 to 18.74, so it points at moderate contamination at the station S1, but very high contamination at the remaining sites (S2–S4) (Tables 3, 11). The contamination factors designated for other metals at the station S1 were < 1, and the contamination degree calculated for this position (*C*_d_ = 5.24) signals a low contamination (Tables 3, 11). The contamination factors (*C*_f_) for points S2–S4 classify the sediments of Straszyn Lake as lowly contaminated in the case of Zn, Pb, and Cd (1 < *C*_f_ ≤ 3), but as moderately contaminated in the case of Cu and Ni (3 < *C*_f_ ≤ 6) (Tables 3, 11). Due to the Cr concentration the sediments from the station S3 were classified as considerably contaminated (16 < *C*_d_ ≤ 32) and from the stations S2 and S4 as very highly contaminated (*C*_d_ > 32) (Tables 3, 11). Thus, Cr played a leading role in the contamination of the examined sediments, which is well presented by the analysis of the value of contamination degree (Fig. 3).

**Table 11:** Contamination factors, contamination degrees, and pollution load index values of metals in sediment of Straszyn Lake

Sampling point	Zn	Cu	Pb	Ni	Cr	Cd	*C*_d_	PLI
	*C*_f_							
1	0.45	0.76	0.33	0.29	2.86	0.54	5.24	0.61
2	1.66	4.41	2.16	5.56	16.77	1.95	32.50	3.18
3	1.52	4.41	3.09	4.48	16.57	1.65	31.71	3.12
4	1.77	4.08	2.33	5.24	18.74	1.60	33.74	3.14

#### Geo-accumulation Index

The *I*_geo_ values (calculated using geochemical background as for PLI) for the study area are presented in Table 12. According to the classification given in Table 4 the calculated values of *I*_geo_ for Zn and Cd in the sediments belong to class 0 and 1, indicating that the sediments were “practically unpolluted” or “unpolluted to moderately polluted.” The values of *I*_geo_ for Cu, Pb, and Ni classified the sediments from “practically unpolluted” to “moderately polluted” (contamination levels 0–2). However, the sediments were “strongly to very strongly polluted” by Cr (level 5), except the samples collected at the station S1 (*I*_geo_ = 0.87, level 1).

**Table 12:** Geo-accumulation index assessment data of metals in sediments and their class

Sampling point	Zn	Cu	Pb	Ni	Cr	Cd
	*I*_geo_					
S1	− 1.77	− 1.03	− 2.43	− 2.81	0.87	− 1.65
S2	0.14	1.54	0.52	1.88	4.13	0.30
S3	− 0.05	1.54	1.01	1.50	4.08	0.05
S4	0.21	1.43	0.62	1.79	4.27	0.06
Level	0–1	0–2	0–2	0–2	1–5	0–1

#### Sediment Quality Guidelines

The concentration of metals in the sediment samples were compared with the consensus-based TEC and PEC values (Table 13). The results show that PEC value was exceeded only in the case of Cr (75% samples); Pb, Ni, and Cd were between TEC and PEC for 9.4%, 21.9%, and 28.1% of the samples, respectively. The concentration of chromium in sediments for most samples exceeds the value, which exhibits an adverse effects on ecosystem. The mean PECQ can be used to predict potential toxicity of sediments as a mixture of contaminants (Ingersoll et al. 2001; Li 2014). The lowest value of PECQ of six metals was found at the station S1 (in the inlet zone): PECQ = 0.06. At the other stations, mean values of PECQ ranged from 0.45 to 0.49. It means that all the sediment samples were potentially nontoxic (PECQ < 0.5) (MacDonald et al. 2000).

**Table 13:** Comparison between sediment quality guidelines and metal concentration of samples in Straszyn Lake

	% of samples < TEC	% of samples between TEC-PEC	% of samples > PEC
Zn	100	0	0
Cu	100	0	0
Pb	90.6	9.4	0
Ni	78.1	21.9	0
Cr	25	0	75
Cd	71.9	28.1	0

#### Potential Ecological Risk Index

The calculated values of PERI for sediments are summarized in Table 14, and the classification of PERI is presented in Table 6. The *E*_r_ values of Zn, Cu, Pb, Ni, and Cr indicated low pollution in the sediment samples. Moderate potential ecological risk (*E*_r_ > 40) was noted in the case of Cd at the sites 2–4. Overall, the mean PERI for metals in the sediment of Straszyn Lake showed low pollution (PERI < 150). The PERI was classified as moderately polluted only at the site 2: PERI = 154.23. When comparing the potential ecological risk index for individual metals (mean values of *E*_r_) with the grade classification, the *E*_r_ was in the order: Cd > Cr > Ni > Cu > Pb > Zn.

**Table 14:** Potential ecological risk index for the sediments

Sampling point	Zn	Cu	Pb	Ni	Cr	Cd	
	*E*_r_						PERI
S1	0.45	3.81	1.65	1.46	5.73	16.31	29.40
S2	1.66	22.04	10.79	27.78	33.55	58.41	154.23
S3	1.52	22.05	15.43	22.41	33.14	49.39	143.94
S4	1.77	20.40	11.65	26.18	37.47	47.89	145.35

#### Chemical Speciation Profile and Mobility Risk of Metals

**Fig. 4:**
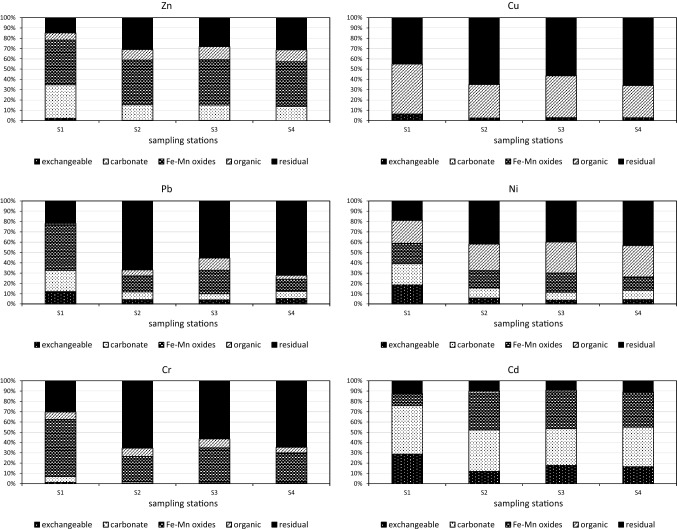
Percentage of each fraction of Zn, Cu, Pb, Ni, Cr, and Cd in the bottom sediments for sampling points of Straszyn Lake

The mean percentages of five sequential extraction fractions (such as exchangeable, bound to carbonates, bound to Fe–Mn oxides, bound to organic matter and residual) for each metal in sediment are illustrated in Fig. 4. The chemical partitioning of metals is described at the stations S2–S4 and separately at the station S1, because the chemical speciation of metals showed rather similar distribution patterns within sampling points, except sampling point S1 (the inlet zone).

*The stations S2*–*S4* Zn was mainly bound to fractions III (Fe–Mn oxides phase, 43.3–43.9%) and V (residual phase, 28.1–31.3%). Similar observations were also made by Sobczynski and Siepak (2001), Wojtkowska et al. (2016), and Zhang et al. (2017). Cu remained mainly associated with fraction V (56.5–60.0%) and with fraction IV (organic, 31.4–40.6%). High amount of Cu in the residual fraction was consistent with the result of Madeyski et al. (2009), Palleiro et al. (2016) and Zhang et al. (2017). The high percentage of the organic fraction in Cu binding was similar to other previous studies of aquatic sediments (Wojtkowska et al. 2016; Zhang et al. 2017) because of the easy formation of high stability constants of organic-Cu compounds (Stumm and Morgan 1981). The high affinity of Cu^2+^ ions for soluble organic ligands may cause an increase in their mobility in sediments (McLean and Bledsoe 1992; Yu et al. 2001; Li et al. 2009). Pb was present, above all, in fraction V (55.4–72.1%) and in fraction III (11.6–22.8%). A similar share of the residual fraction in binding of lead was observed in mainstream, tributaries, and lakes of the Yangtze River catchment of Wuhan (Yang et al. 2009), in the Mahandi River basin in India (Sundaray et al. 2011), and in small water reservoirs in Poland (Madeyski et al. 2009). A high amount of Pb in the Fe–Mn oxides fraction was observed in the Pearl River Estuary in China (Zhang et al. 2017). Ni associated with fraction V constituted between 39.9% and 43.3%, and with fraction IV between 25.6% and 30.6%. Cr preferentially bound to fraction V (56.5–65.4%) and to fraction III (24.7–33.5%). The significant role of the residual fraction in binding of Cr and Ni was consistent with the result of Singh et al. (2005), Szarek-Gwiazda et al. (2011), Baran and Tarnawski (2015), and Zhang et al. (2017) and suggests that these two metals were strongly bound with crystalline structures of the minerals (Xiao et al. 2015). Cd was mainly bound to fraction II (carbonates phase, 35.7–40.2%) and fraction III (33.3–35.7%). The high percentages of carbonate fraction in Cd binding also was observed by Singh et al. (2005) and Farkas et al. (2007). This may result from the high concentration of HCO_3_^−^ in the sediments (Yang et al. 2009). This fraction is sensitive to environmental conditions, such as pH. A reason for Cd pollution may be Cd binding to carbonates, when pH decreases (Balistrieri et al. 2007). Cd associated with the fraction I (exchangeable) constituted between 11.9% and 17.8%. These values were associated with the strong adsorption of Cd onto colloids in the sediments (Yang et al. 2009). The crucial contribution of the first three fractions in Cd binding (exceeded 85% in the present study) agreed with that obtained from the sediments in many lakes and rivers around the world (Lopez-Sachez et al. 1996; Jain 2004; Njeng et al. 2009; Sundaray et al. 2011; Wojtkowska et al. 2016). Except Cd, metals binding with the exchangeable fraction were weak and depended on a metal type ranging between 0.2% and 5.7%. Zn associated with the carbonate constituted between 13.7% and 15.2% and obtained lower values than the associated Cd but higher than other metals (0.1–9.8%).

*Station S1* Zn remained mainly associated with the carbonate fraction and the Fe–Mn oxides fraction (32.4% and 43.3%, respectively), Cu with the organic fraction and the residual fraction (48.4% and 45.2%, respectively), Pb with the fractions: carbonate, Fe–Mn oxides and residual (20.6%, 44.4% and 21.9% respectively). Ni was bound to each fraction in approximately 20% (from 18.3 to 22.1%), Cr to the Fe–Mn oxides and residual fractions (55.6% and 30.4%) and Cd to the exchangeable and carbonate ones (28.6% and 46.9%). Overall, all the examined metals at the station S1 were bound with mobile fractions (the exchangeable, carbonate and Fe–Mn oxides) in higher degree than at the other stations. These results indicate that the speciation of metals in the sediments from the inlet zone of Straszyn Lake showed higher bioavailability compared to the sediments from the middle part of this reservoir.

In summary, the distributions of metals (mean values) were as follows:

- Zn: Fe–Mn oxides > Residual > Carbonate > Organic > Exchangeable
- Cu: Residual > Organic > Exchangeable > Fe–Mn oxides > Carbonate
- Pb: Residual > Fe–Mn oxides > Organic ≈ Carbonate > Exchangeable
- Ni: Residual > Organic > Fe–Mn oxides > Carbonate > Exchangeable
- Cr: Residual > Fe–Mn oxides > Organic > Carbonate > Exchangeable
- Cd: Carbonate > Fe–Mn oxides > Exchangeable > Residual > Organic

The data presented above indicate that Cu, Pb, Ni and Cr were characterized by the predominance of the residual fraction, but Zn and Cd were strongly bound with labile fractions (the Fe–Mn oxides fraction and the carbonate fraction, respectively). In general, the metals in the sediments of Straszyn Lake were bound to different fractions in different degree. Thus, the RAC was utilized to assess the risk connected with the presence of metals in an aquatic environment. Metals occurring in the exchangeable and carbonate fractions are mobile and available to living organisms and thereby toxic to them (Du Laing et al. 2009), so the risk assessment code is an efficient tool for estimation of the risk connected with the release of metals from the sediments. The mean percentage of metals in the exchangeable and carbonate fractions decreased in following order:

- Cd (59.1%) > Zn (19.8%) = Ni (19.8%) > Pb (16.6%) > Cu (3.3%) > Cr (2.7%)

According to RAC classification (Table 7), it was found to be a very high risk for Cd release from bottom sediments, medium risk for Zn, Ni, and Pb release, and low risk in case of Cu and Cr. Sundaray et al. (2011) and Ke et al. (2017) based on RAC also found that the highest mobility of Cd posed a higher environmental risk. Low bioavailability of Cr in contrast to Pb, Cd, and Zn was in turn observed by Ciazela et al. (2018) in bottom sediments of an urban zone-river-oxbow lake system in the Middle Odra Valley.

### Correlation Coefficient Analysis

Correlations among metals in sediments provide information on the origin and migration of these elements (Dragovic et al. 2008; Bhuiyan et al. 2010; Suresh et al. 2011). The results of the Pearson correlation analysis are presented in Table 15. A significantly positive correlation (*p* < 0.001) exist between all metal pairs. The highest positive correlation were found between Zn–Cu (0.911), Zn–Cr (0.947), Cu–Ni (0.914), Cu–Cr (0.932), and Ni–Cr (0.915). The very strong correlation among metals indicates their common origin (Ke et al. 2017, Bhuyan et al. 2017, Ciazela et al. 2018). However, Broda and Frankowski (2017) observed in the Lednica Lake (in medieval waterlogged oak wood, Wielkopolska Region, Poland) that Cd, Ni, and Pb originated from run-off to the lake from the drainage basins, whereas Cu and Zn from the natural weathering processes. Kuriata-Potasznik et al. (2016) also found that the sources of Cd, Ni, and Pb in the Symsar Lake (Northern Poland) were anthropogenic. The authors pointed out that sedimentation process can limit the dispersion of metals outside the aquatic ecosystem. In addition there was a positive correlation (*p* < 0.001) between all metals and fine fractions of sediments: clay silty and clay (0.682 < *r* < 0.996) but a negative correlation between all metals and sand fraction. Similar results were obtained by Szarek-Gwiazda et al. (2011), Strzebońska et al. (2015), and Czaplicka et al. (2016). The particulate fractions could play a significant role in binding metals due to their large specific and high adsorption capacity, which decreases with increasing grain size (Hu et al. 2013). Ciazela et al. (2018) noted that metals associated with the finest granulometric fractions of the bottom sediments seem to be less bioavailable in relation to the metals in the larger fractions, which can reduce their impact on the environment. The organic matter content was not significantly correlated with any of the metals except Pb (0.453, *p* < 0.01). This result agreed with those of Ke et al. (2017) and indicated that the distribution of Pb was controlled by the organic matter content in the sediments. A significantly negative correlation was observed between pH and Zn (− 0.459, *p* < 0.01), Ni (− 0.367, *p* < 0.05), and Cr (− 0.448, *p* < 0.05), so the pH may affect the distribution of Zn, Ni, and Cr in the sediments of Straszyn Lake.

**Table 15:** Pearson correlation analysis among total contents of metals and sediment physicochemical properties

	Zn	Cu	Pb	Ni	Cr	Cd	Sand	Clay silty	clay	pH	Organic matter
Zn	1										
Cu	0.911*	1									
Pb	0.860*	0.894*	1								
Ni	0.889*	0.914*	0.833*	1							
Cr	0.947*	0.932*	0.876*	0.915*	1						
Cd	0.793*	0.705*	0.731*	0.722*	0.760*	1					
Sand	− 0.985*	− 0.957*	− 0.918*	− 0.957**	− 0.994*	− 0.902*	1				
Clay silty	0.986*	0.968*	0.930*	0.962*	0.996*	0.915*	− 0.999*	1			
Clay	0.874*	0.755*	0.708*	0.810*	0.875*	0.682*	− 0.909*	0.892*	1		
pH	− 0.459**	ns	ns	− 0.367***	− 0.448***	ns	0.510**	− 0.475**	− 0.821*	1	
Organic matter	ns	ns	0.453**	ns	ns	ns	− 0.397***	0.380***	0.524**	− 0.529**	1

*ns* not significant

*p < 0.001; **p < 0.01; ***p < 0.05

## Conclusions

In this study, the metals pollution of the sediments of Straszyn Lake was evaluated using different tools, methods, and indices. The mean concentrations of all the metals in the sediments of the study area were higher than the geochemical background. In particular, the concentrations of Cr, Cu, and Ni in samples of the middle part of Straszyn Lake indicate that human activities had a direct effect on the content of metals in sediments. The pollution load index (PLI) derived from contamination factors showed that, due to Cr concentration, sediments were moderately polluted to moderately-highly polluted. The assessment of *I*_geo_ also indicated that the sediments were strongly to very strongly polluted with Cr. However, depending on the choice of geochemical background, all the indices based on this value can be manipulated to show very different results. To predict the biological toxicity of the metals sediments quality guidelines (SQGs), potential ecological risk index (PERI) and risk assessment code (RAC) were introduced. According to mean probable effect concentration quotients (PECQ) the sediments were potentially nontoxic, but Cr was likely to often exhibit an adverse effects on the ecosystem. On the other hand, potential ecological risk index (PERI) values suggested that Cd was the most important factor affecting the ecological environment of Straszyn Lake. The RAC confirmed these results due to the crucial contribution of labile fractions in Cd binding. The very strong correlation calculated between all the metals indicated their common origin.
